# European economic crisis and health inequities: research challenges in an uncertain scenario

**DOI:** 10.1186/s12939-014-0059-5

**Published:** 2014-07-25

**Authors:** Antonio Escolar-Pujolar, Amaia Bacigalupe, Miguel San Sebastian

**Affiliations:** 1 Delegación Territorial de Igualdad, Salud y Políticas Sociales, Junta de Andalucía, Cádiz, Spain; 2 Department of Sociology, University of the Basque Country (UPV/EHU), Leioa, Spain; 3 Epidemiology and Global Health, Department of Clinical Medicine and Public Health, Umeå university, Umeå, Sweden

**Keywords:** Health inequities, Economic crisis, Europe

## Abstract

**ᅟ:**

## 

The economic and social crisis that the European population has experienced since 2008 has fuelled interest in the study of its potential health impacts, especially when it comes to the more vulnerable southern countries like Greece, Portugal, Italy and Spain [[1],[2]]. With regard to public health, and particularly social epidemiology, the need to deal with the problem of the economic crisis encourages natural experiments from which it is possible to visualize the relationships between social determinants and health, and to assess the usefulness of the health policies and interventions required to buffer the negative impact of the crisis.

Since the 1929 stock market crash in the United States, there have been different episodes of economic crises that have affected almost every continent, with diverse health consequences [[3]]. Unlike previous economic crisis, this one is directly affecting European countries that are included in the group of so-called developed countries and that are located in the centre of the global economic system. Measures implemented by the respective European governments to overcome the crisis have been similar to those used in previous crisis that affected countries of the global South. The delivery of financial assistance from the International Monetary Fund (IMF) and the European Central Bank (ECB), primarily aimed at preventing the collapse of the European banking system, has been dependent on the compliance with structural adjustment reforms by the governments of all the ‘rescued countries’. These measures have mainly focused on the implementation of public spending cuts, as well as privatization of public services and a deregulation of markets. Despite the resistance of social movements and the recognition by the IMF itself of their own errors in the management of the Greek debt crisis, these adjustments continued to be imposed [[4],[5]].

One of the most immediate consequences of the crisis has been the sharp increase in unemployment, which has spiked particularly among young people. When it comes to health, there is a need to understand the implications of unemployment in this age group and to study the effects it may have in the short, medium and long term. Spain, together with Greece, has led the European unemployment statistics since 2008, both overall (26.1%) and among people under 25 (56.0%) [[6]]. Several studies have already highlighted the relationship between the economic crisis and mental health problems such as a higher frequency of episodes of anxiety and depression, as well as suicide in these countries [[7],[8]].

The increase in social inequality constitutes one of the major side effects of the implementation of structural adjustment policies of neoliberal orientation [[9]]. The concentration of wealth, the increasing rate of relative poverty, the growing social exclusion of groups such as young people, immigrants and ethnic minorities, the expansion of precarious forms of employment, the cuts in social spending, and the policies directed to hinder an equitable access to educational resources are among the most important elements of the current package of policies. The cuts in funding and the continuous privatization plans of hospitals and primary care centers are also causing progressive deterioration in the quality of services, in waiting times as well as promoting co-payments of drugs and health care services. In addition, the exclusion of non-regularized immigrants from the public health care services should also be considered as a negative consequence of the policies put in place as a result of the economic crisis [[10]]. Despite all the evidence accumulated over the years about the negative health consequences of austerity in public expenditure, these policies continue to be applied [[11]].

## Limitations of published studies and the need for new knowledge

Much of the literature on the relationship between economic crises and health, concerning both the current and the previous crises, has been primarily focused on measuring the impact on overall mortality and on specific causes such as communicable diseases, external causes and suicides. These have generally been descriptive and quantitative studies, mainly ecologically based and referring to a single country in which the magnitude of mortality is compared before and after a crisis. Studies have also examined changes in some proximal health determinants, such as the consumption of alcohol and tobacco or physical exercise. However, the consideration of both structural determinants and political and ideological factors in the analyses has been very limited, despite their importance in the definition of macroeconomic conditions from which the opportunities for achieving better or worse health statuses are created [[12],[13]]. Studies exploring changes in the incidence of chronic diseases, in the accessibility to health services, in the coverage of health prevention programmes, professional practice, or in health care services management schemes and budgetary adjustments are rare.

The inequality dimension has also been a neglected aspect in this research area. Most of the studies have considered the entire population as a unit of analysis without taking into account the variability in the frequency and characteristics of health problems according to social position (i.e.: ethnicity, class, gender, educational level, occupation, etc.). Studies evaluating the impact of economic and social policies on social inequalities in health constitute an area of special interest where the IJEH is of course willing to prioritize its publications. It is equally important to assess the impact of policies that support free and universal access to public services, maintain social expenditure and promote economic aid to unemployed people, in terms of cushioning and reducing health inequalities. Further investigations are needed on the impact of the crisis on child and maternal health, since the impacts of structural adjustment measures on the health of pregnant women, fetuses and children in the early years of the life are critical to assess the risks of health problems in adulthood [[14]].

Research into the relationships between economic crises and health should not be designed solely for the purpose of identifying increases in the frequency or intensity of health problems. As noted in some studies, the changes imposed by the economic crises can also have positive aspects regarding population health, such as those which were observed in the special transition period among the population of Cuba after the implosion of the USSR in 1989 [[15]] or the decrease in traffic mortality rates due to a lower use of vehicles [[2]]. This does not imply that, even if certain positive effects in terms of population health from the current crisis would be found, it would be beneficial to recommend structural adjustment policies [[16]].

In relation to the research focus of this thematic series, sociological and anthropological analyses of socio-economic inequalities in health related to the economic crisis could bring about new elements of knowledge. Research is needed on individuals’ experience and perceptions of the effects of the crisis in everyday life within the most affected groups, such as youth, families evicted from their homes, those affected by employment layoffs or the long-term unemployed. In addition, individual and collective responses to the crisis that determine different health outcomes are influenced by cultural contexts. This involves exploring issues such as the role that social support networks play at the family, group or institutional level, and how these would be helpful in the defense of social models in which solidarity is a central value. In this light, micro-sociological studies are essential to finding answers and could help to explain some of the different health impacts observed between countries.

In the context of the current crisis we consider the investigation of possible paradoxical effects on the public health of the European population to be of great interest. This information could be crucial for the definition of public health policies that need to be implemented during the transition to economic de-growth [[17]], an alternative to the dominant development model based on the unlimited growth of Gross Domestic Product (GDP), and which offers a higher guarantee for the sustainability of human life [[18]].

The opportunities for improvement of the scientific knowledge offered by the current economic crisis must be taken into account by researchers interested in public health, not only those who are examining the current crisis, but those who are examining longer term prospects with the purpose of helping build more equitable societies with regard to health. This is what encourages our presence as editors in this thematic series on the European economic crisis, organized by IJEH, and to which we invite all the authors who share this desire to contribute in the coming years.

In this first cluster of papers, we will travel from the North to the South of Europe with stopover in Greece. During the journey, diverse methodological approaches and different health outcomes have been used to examine the health impacts of the economic crisis. De Vogli gives us a broad perspective on the origins of the economic crisis, summarizes some of its terrible health consequences and points out the need for a new global political strategy that abandons the current neoliberal policies and creates an economy that serves the humankind. In the north, Blomqvist et al. observe an increase in women’s mental distress in four labour market groups in the county of Stockholm while Ásgeirsdóttir et al. show an increase in the health-income inequality favoring the better off in Iceland. A comparison of pharmaceutical sector policies implemented in Finland and Portugal to contain country spending during the economic recession are analysed by Leopold et al. In southern Spain, a rapid increase in suicide attempt rates and the association of unemployment with growing suicidal behaviour in men is described by Córdoba-Dona et al. and Kyriopoulos et al present current inequalities in access to health care services for chronic patients in Greece. Finally, Bacigalupe and Escolar end the cluster with a review about how much is known in the literature about the impact of economic crises on social inequalities in health.
